# The Nuclear-Localized RxLR Effector PvAvh74 From *Plasmopara viticola* Induces Cell Death and Immunity Responses in *Nicotiana benthamiana*

**DOI:** 10.3389/fmicb.2019.01531

**Published:** 2019-07-10

**Authors:** Xiao Yin, Boxing Shang, Mengru Dou, Ruiqi Liu, Tingting Chen, Gaoqing Xiang, Yanzhuo Li, Guotian Liu, Yan Xu

**Affiliations:** ^1^State Key Laboratory of Crop Stress Biology in Arid Areas, College of Horticulture, Northwest A&F University, Yangling, China; ^2^Key Laboratory of Horticultural Plant Biology and Germplasm Innovation in Northwest China, Ministry of Agriculture, College of Horticulture, Northwest A&F University, Yangling, China

**Keywords:** downy mildew, oomycete, RxLR effector, cell death, plant immunity

## Abstract

Downy mildew is one of the most serious diseases of grapevine (*Vitis* spp). The causal agent of grapevine downy mildew, *Plasmopara viticola*, is an obligate biotrophic oomycete. Although oomycete pathogens such as *P. viticola* are known to secrete RxLR effectors to manipulate host immunity, there have been few studies of the associated mechanisms by which these may act. Here, we show that a candidate *P. viticola* RxLR effector, PvAvh74, induces cell death in *Nicotiana benthamiana* leaves. Using agroinfiltration, we found that nuclear localization, two putative *N*-glycosylation sites, and 427 amino acids of the PvAvh74 carboxyl terminus were necessary for cell-death-inducing activity. Using virus-induced gene silencing (VIGS), we found that PvAvh74-induced cell death in *N. benthamiana* requires EDS1, NDR1, SGT1, RAR1, and HSP90, but not BAK1. The MAPK cascade components MEK2, WIPK, and SIPK were also involved in PvAvh74-induced cell death in *N. benthamiana*. Transient expression of PvAvh74 could suppress *Phytophthora capsici* colonization of *N. benthamiana*, which suggests that PvAvh74 elicits plant immune responses. Suppression of *P. capsici* colonization also was dependent on nuclear localization of PvAvh74. Additionally, PvAvh74-triggered cell death could be suppressed by another effector, PvAvh8, from the same isolate. This work provides a framework to further investigate the interactions of PvAvh74 and other RxLR effectors with host immunity.

## Introduction

Plants are subject to attack by various pathogens, and to prevent pathogen invasion, they have developed a two-tier, innate immune system (Jones and Dangl, 2006). The first-tier immunity is activated by pattern recognition receptors (PRRs), which recognize specific molecules secreted by the pathogen. Such molecules are termed pathogen/microbe-associated molecular patterns (PAMP/MAMPs), and include fungal chitin and bacterial flagellin at the plasma membrane (Nürnberger et al., 2004; Bittel and Robatzek, 2007; Naito et al., 2008). This recognition is a basal immune mechanism and is termed PAMP-triggered immunity, or PTI (Jones and Dangl, 2006). However, some pathogens can secrete effectors to break through PTI. For many plants, second-tier immunity is related to resistance proteins, encoded by *R* genes, which recognize the effectors and initiate the hypersensitive response (HR) (Dodds and Rathjen, 2010). This is called effector-triggered immunity (ETI) (Jones and Dangl, 2006).

The effectors recognized by plant *R* genes are also defined as avirulence (*Avr*) genes. In recent years, several *Avr* genes have been identified in *Phytophthora* species. For example, *Avrblb1*, *Avrblb2*, *Avr2*, *Avr3a*, and *Avr4* have been identified in *Phytophthora infestans* (Armstrong et al., 2005; van Poppel et al., 2008; Vleeshouwers et al., 2008; Oh et al., 2009; Gilroy et al., 2011), and *Avr1b*, *Avr1a/3a*, *Avr3b*, *Avr3c*, *Avr4/6*, and *Avr5* have been identified in *Phytophthora sojae* (Shan et al., 2004; Dong et al., 2009, 2011a,b; Qutob et al., 2009; Dou et al., 2010). All of these Avr proteins contain signal peptides and conserved RxLR-dEER motifs at their amino (N) termini, and are defined as Arg-x-Leu-Arg (RxLR) effectors.

The best-studied effectors in oomycete pathogens are probably the cytoplasmic RxLR and Crinkler (CRN) effectors (Jiang and Tyler, 2012). In addition to the *Avr* genes, these effectors can also trigger cell death in plant leaves. For example, an RxLR effector from *P. sojae*, Avh241, triggers cell death in leaves of *N. benthamiana* and *Solanum lycopersicum*. Avh241-induced cell death requires the mitogen-activated protein kinases (MAPKs), NbWIPK and NbMEK2 (Yu et al., 2012). Another RxLR effector from *P. sojae*, Avh238, triggers cell death in *N. benthamiana*, *Nicotiana tabacum*, *Solanum tuberosum*, *S. lycopersicum*, and *Solanum melongena* leaves. Avh238-induced cell death in tobacco leaves relies on NbMEK2, but not the receptor-like kinases SOBIR1 and BAK1 (Yang et al., 2017). In addition, some CRN effectors, such as the *P. sojae* effector CRN63, can induce cell death, and silencing of CRN63 in *P. sojae* results in reduced virulence in soybean. Other effectors such as CRN8 from *P. infestans* (van Damme et al., 2012), or CRN4 (Mafurah et al., 2015) and CRN83_152 from *P. capsici*, can also induce cell death in tobacco (Stam et al., 2013).

Studies of pathogenicity of oomycetes have been greatly facilitated by genome sequencing of many species. Genome-sequenced species include *P. infestans* (responsible for late blight of potato) (Haas et al., 2009), *Phytophthora nicotianae* (tobacco black shank) (Liu et al., 2016), *P. sojae* (soybean root rot), *Phytophthora ramorum* (sudden oak death) (Tyler et al., 2006), *P. capsici* (root and fruit rot of pepper and cucurbits) (Lamour et al., 2012), and *Phytophthora ultimum*, responsible for damping off and root rot in a wide range of hosts (Lévesque et al., 2010). To date, obligate biotrophic oomycetes cannot be artificially cultured, making their study difficult. Genome sequences are also available for several biotrophic downy mildew pathogens including *Hyaloperonospora arabidopsidis* (Baxter and Tyler, 2010), *Pseudoperonospora cubensis* (Burkhardt et al., 2015), and *Plasmopara halstedii* (Sharma et al., 2015).

Another biotrophic downy mildew pathogen, *Plasmopara viticola* (Berk and Curt.; Berlese and de Toni), causes grapevine downy mildew, a serious disease of grapevine. However, little is known about *P. viticola* effectors and how these proteins interact with the grapevine host. Several *P. viticola* effectors were identified from cDNA sequencing of *in vitro* germinated zoospores. These included putative secretory hydrolases and RxLR effectors (Mestre et al., 2012). In addition, 51 RxLR effectors and 10 CRN effectors were predicted from RNA-sequencing analysis of grapevine leaves infected by *P. viticola* isolates “ZJ-1-1,” “JL-7-2,” and “CSIRO-L-2” (Yin et al., 2015). *PvRxLR28*, a *P. viticola* RxLR effector from isolate “ZJ-1-1,” was studied in depth. The *PvRxLR28* gene was highly expressed at 6 h post-infection (hpi), and showed broad cell death suppression activity and enhanced *P. viticola* and *Phytophthora parasitica* leaf colonization (Xiang et al., 2016). In a subsequent study, a candidate RxLR effector, PvRxLR16, was identified and reported to cause cell death or immune responses in tobacco leaves. The cell death induced by PvRxLR16 required the G-two allele of Skp1 (SGT1), Mla12 resistance (RAR1), and heat shock protein 90 (HSP90), but not the receptor-like kinase SERK3/BAK1 (Xiang et al., 2017). More recently, 83 candidate RxLR effectors were cloned from a Chinese *P. viticola* isolate and preliminarily analyzed. Among these candidate RxLR effector genes, 10 triggered cell death in *N. benthamiana* (Liu et al., 2018). Recently, an isolate of *P. viticola* from Italy, “PvitFEM01,” was sequenced. Finally, an RxLR effector, PVITv1008311, was described as an elicitor that could trigger cell death in the wild species *Vitis riparia in vitro*, but not in *Vitis vinifera in vitro*. This effector has potential to be used as a marker to screen grapevine varieties for potential resistance (Brilli et al., 2018).

Here, we characterize a candidate RxLR effector, designated PvAvh74 (*P. viticola* avirulence homolog), from the *P. viticola* isolate “YL.” PvAvh74 induced cell death in *N. benthamiana* leaves, and this involved MAPK cascades and several key proteins of plant immunity. Transient expression showed that PvAvh74-triggered cell death depended on nuclear localization and two putative *N*-glycosylation sites. Transient expression of PvAvh74 induced ROS accumulation and disease resistance against *P. capsici* in *N. benthamiana*. PvAvh74-triggered cell death could be suppressed by another effector, PvAvh8, from the same isolate.

## Materials and Methods

### Plant and Fungal Materials and Growth Conditions

*Nicotiana benthamiana* plants were cultivated at 25/20°C in a greenhouse with a photoperiod of 18 h light/6 h dark. *V. vinifera* susceptible cultivar Thomson seedless was cultured *in vitro* on half-strength Murashige Skoog (MS) medium containing 30 g/L sucrose and 7 g/L as described by Brilli et al. (2018) with some modification. The grapes *in vitro* were cultivated at 25°C in a tissue culture room with a photoperiod of 18 h light/6 h dark. *P. capsici* infection assays were carried out according to Song et al. (2015). Briefly, mycelia were grown on 10% V8 juice agar medium at 25°C in the dark. Zoospores were subsequently induced by incubation at 4°C. The suspension concentration of zoospores was 1.0 × 10^4^/ml as determined using a hemacytometer.

### Vector Construction

All primers used in this study are listed in Supplementary Table S1. A schematic diagram of the vector constructs is shown in Supplementary Figure S1. The *PvAvh74* gene was amplified from genomic DNA from *P. viticola* isolate “YL” (Yin et al., 2017). For the PVX assay, PvAvh74 without the predicted signal peptide and PvAvh74 deletion mutants were cloned and subsequently ligated into the *SmaI* and *NotI* restriction enzyme sites of the pGR107 vector. The nuclear export signal (NES) sequence and corresponding mutated sequence (nes) (Wen et al., 1995) were extended to the carboxyl (C) terminus of *PvAvh74* using overhanging primers (Supplementary Table S1). Then, *PvAvh74*, *PvAvh74*^NES^, and *PvAvh74*^nes^ were ligated into the pCAMBIA2300-GFP vector cut using appropriate restriction enzymes. For the virus-induced gene silencing (VIGS) assay, fragments of target genes were amplified and fused into the *EcoRI* and *BamHI* restriction enzyme sites of the pTRV2 vector. To confirm the secretion function of the PvAvh74 signal peptide, the sequence was cloned and then introduced into pSUC2T7M13ORI (pSUC2) (Jacobs et al., 1997) cut using *EcoRI* and *XhoI* restriction sites.

### *Agrobacterium*-Mediated Transient Expression

Constructions with verified sequence were transformed into *Agrobacterium tumefaciens* strain GV3101 by electroporation. The recombinant strains were cultured in Luria-Bertani (LB) medium containing appropriate antibiotics for 48 h at 28°C with shaking at 200 rpm. *A. tumefaciens* cells were harvested by centrifugation (3000 × *g*, 5 min), washed three times in 10 mM MgCl_2_, and resuspended in infiltration buffer (10 mM MgCl_2_, 10 mM MES, pH 5.7, and 150 μM acetosyringone). The suspension was diluted to an OD_600_ of 0.4 and cultured in the dark at 28°C for 3 h. Then, the suspension was infiltrated into plant leaves using a needleless syringe.

### Confocal Microscopy

The *PvAvh74* gene and mutant derivatives were cloned into the pCAMBIA2300-GFP vector to generate the vector PvAvh74/PvAvh74^NES/nes^-GFP (Supplementary Figure S1). The full-length sequence of *AtHY5* (*ELONGATED HYPOCOTYL 5*; AT5G11260) was cloned into the pBI221-mCherry vector to generate AtHY5-mCherry. Sequence-verified constructions were introduced into *N. benthamiana* protoplasts using a PEG-calcium transfection method (Yoo et al., 2007; Ma et al., 2018). The transformed *N. benthamiana* protoplasts or agroinfiltrated leaves were observed using confocal microscopy (TCS SP8, Leica, Germany). The excitation wavelengths of green fluorescent protein (GFP) and mCherry were 488 and 587 nm, respectively.

### Virus-Induced Gene Silencing Assays

Virus-induced gene silencing assays were performed as described by Senthil-Kumar and Mysore (2014). Briefly, plasmid TRV1 and the derived plasmid TRV2 were introduced into *A. tumefaciens* GV3101 by electroporation. *Agrobacterium* suspensions of TRV2 and TRV1 were mixed at a 1:1 ratio and adjusted to an OD_600_ of 0.1, and then infiltrated into *N. benthamiana* leaves using a needleless syringe. The control plants were infiltrated with TRV2:GFP or TRV2:PDS. Three weeks later, when the leaves of PDS-silenced plants showed photobleaching (Supplementary Figure S2), the silencing efficiencies of the target genes were evaluated by quantitative real-time PCR (qRT-PCR). Finally, the treated plants were prepared for cell death assays.

### Yeast Signal Sequence Trap System

The secretion function of the PvAvh74 signal peptide was validated using a yeast secretion assay according to Jacobs et al. (1997). Briefly, recombinant pSUC2 plasmids were transformed into yeast strain YTK12 by the lithium acetate method (Gietz et al., 1995). Transformants were first selected on CMD-W medium (without Trp), and then positive clones were plated onto YPRAA medium for the invertase secretion assay. The reduction of TTC to insoluble red colored triphenylformazan was monitored to detect secreted invertase activity (Oh et al., 2009).

### RNA Isolation and RT-PCR Analysis

Total RNA was extracted from *N. benthamiana* leaves using the Omega Plant RNA Kit (Omega Bio-tek, United States) according to the manufacturer’s protocol. First-strand cDNA was synthesized using PrimeScript RT reagent with gDNase (TaKaRa, Biotechnology; Dalian, China) following the recommended protocol. RT-PCR using SYBR Premix Ex Taq (TaKaRa) was carried out as described previously by Li et al. (2017). The *NbEF1α* gene was used as an internal control.

To examine expression patterns of *PvAvh74* and *PvAvh8*, *P. viticola* infection was carried out as described previously (Liu et al., 2015). The leaf disks of *V. vinifera* susceptible cultivar Pinot Noir were inoculated with *P. viticola* “YL” (40 μl of sporangia suspension with a concentration of 5 × 10^4^ sporangia/ml). The samples were collected at 0, 6, 12, 24, 48, 72, 96, and 120 hpi for RNA extraction (Liu et al., 2019). Then, RT-PCR was performed as described above.

### Electrolyte Leakage and Histochemistry Assay

Ion leakage resulting from cell death was measured as previously described (Yu et al., 2012). In brief, five leaf disks (diameter, 9 mm) were rinsed three times with distilled water and then soaked in 5 ml of distilled water in a 10-ml tube. The conductivity of the solution was measured after 3 h at room temperature using a conductivity meter (DDS-307; LeiCi, Shanghai, China) to generate value “A.” The tubes containing the solution and leaf disks were then boiled for 25 min. After cooling to room temperature, the conductivity was measured to generate value “B.” Finally, the electrolyte leakage was calculated as the percentage (%) of “A”/“B.” The assay was repeated for a total of three times.

Trypan blue staining was used to observe necrosis according to Xiang et al. (2017). Briefly, agroinfiltrated leaves were boiled in trypan blue solution for 10 min and cooled to room temperature for 12 h. Then, the samples were destained in chloral hydrate solution.

For 3,3′-diaminobenzidine (Sigma-Aldrich, Germany) staining, agroinfiltrated *N. benthamiana* leaves were stained with DAB solution (1 mg/ml) for 10 h at room temperature. The samples were boiled in 95% ethanol for 15 min for decolorizing before being photographed. Relative levels of DAB staining were quantified using Image J software as described by Rajput et al. (2014). For diphenyleneiodonium chloride (DPI) treatment, DPI (Sigma-Aldrich, Germany) was added in *A. tumefaciens* infiltration buffer to a final concentration of 100 μM.

### Western Blotting

Protein extraction and Western blot assays were performed according to Su et al. (2018). Approximately 20 μg of protein was resolved on a 10% sodium dodecyl sulfate (SDS) polyacrylamide gel and transferred onto a polyvinylidene fluoride membrane (PVDF) membrane (Roche, Product No. 03010040001). The membrane was blocked in 5% non-fat dry milk. Then, the membrane was incubated with mouse monoclonal anti-HA antibody (Affinity Biosciences, OH, United States) for 2 h. After washing with PBST (PBS with 0.5% Tween 20) three times, the membrane was incubated with goat anti-mouse IgG (H&L)-HRP polyclonal antibody (Affinity Biosciences, OH, United States). Finally, the membrane was washed five times and visualized using the HRP-ECL system (TransGen Biotech, China).

## Results

### PvAvh74 Triggers Cell Death in *N. benthamiana* Leaves

**FIGURE 1:**
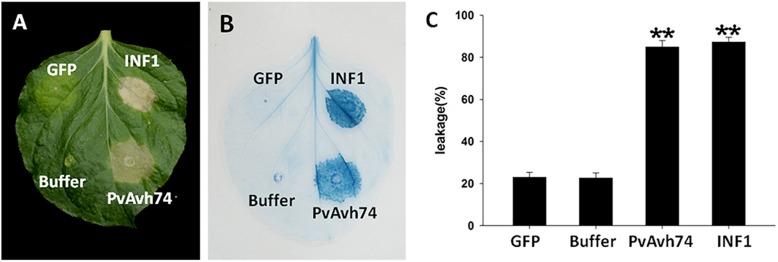
PvAvh74 induced cell death in *Nicotiana benthamiana*. **(A)** PvAvh74 induces necrosis at infiltrated sites in *N. benthamiana* leaves. Images were captured 6 dai. A typical leaf showing necrotic lesions is shown. **(B)** Necrosis induced by PvAvh74 was observed by trypan blue staining. **(C)** Quantification of cell death by measurement of electrolyte leakage. Data are means ± SE (standard error) based on three independent replicates (Student’s *t*-test, ^∗∗^*P* < 0.01).

We previously analyzed the genome of *P. viticola* isolate “YL” (Yin et al., 2017) for genes encoding potential RxLR effectors (unpublished data). We selected one of these, designated *PvAvh74*, to assess its potential effector function *in vivo.* We expressed the *PvAvh74* gene, lacking its predicted signal peptide sequence, transiently in *N. benthamiana* leaves using *Agrobacterium* infiltration. Six days after infiltration (dai), infiltrated sites showed necrosis (Figure 1A). The necrotic lesions appeared similar to those resulting from infiltration of a vector expressing INF1, previously recognized as a positive control for inducing cell death (Kamoun et al., 1998; Figure 1A). To better visualize the symptoms, we stained the infiltrated leaves with trypan blue (Figure 1B). Quantification of cell death was measured by electrolyte leakage (Figure 1C). We also expressed PvAvh74 transiently in *N. tabacum* and grape *in vitro* leaves. Remarkably, expression of PvAvh74 caused necrosis in *N. tabacum*, but not in grape *in vitro* (*V. vinifera* susceptible cultivar Thomson seedless) (Supplementary Figure S3). These results indicated that PvAvh74 can induce cell death in *N. benthamiana* and *N. tabacum* leaves.

### Functional Validation of the Signal Peptide of PvAvh74

The PvAvh74 protein contains an amino-terminal signal peptide (SP) sequence allowing for its secretion. To validate the function of this sequence, we carry out a genetic assay based on the requirement of invertase secretion for yeast cells to grow on sucrose or raffinose media (Oh et al., 2009). The predicted SP sequence of PvAvh74 (N-terminal 20 amino acids) was cloned and fused into the vector pSUC2 (Jacobs et al., 1997). Resultant constructs of PvAvh74-SP and Avr1b-SP (positive control) (Shan et al., 2004) enabled the invertase mutant yeast strain YTK12 to grow on raffinose-containing medium (YPRAA) (Supplementary Figure S4). In addition, invertase secretion of PvAvh74-SP and Avr1b-SP was confirmed to catalyze conversion of 2,3,5-triphenyltetrazolium chloride (TTC) to the insoluble red-colored triphenylformazan. The yeast strain YTK12 and YTK12 carrying the empty pSUC2 vector were used as negative controls (Supplementary Figure S4). These results indicated that the predicted signal peptide of PvAvh74 is functional and that PvAvh74 is likely secreted from *P. viticola*.

### PvAvh74-Induced Cell Death Depends on Nuclear Localization

**FIGURE 2:**
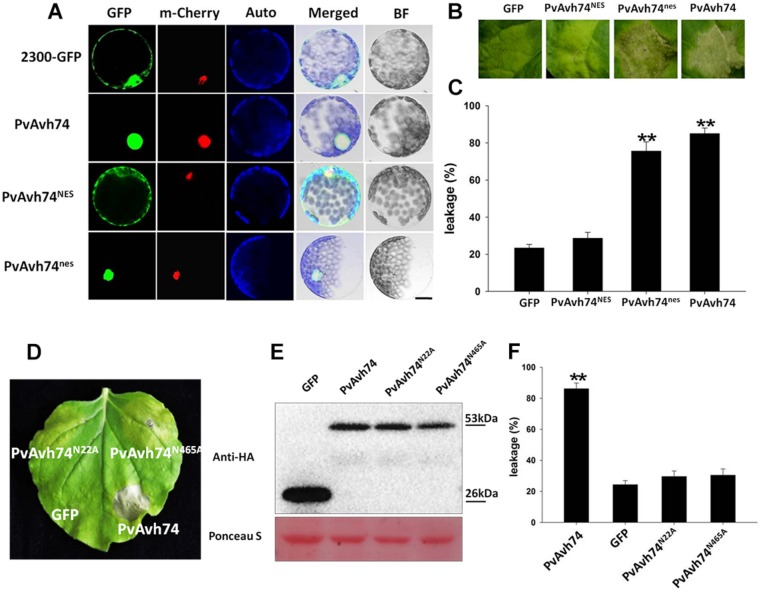
PvAvh74-induced cell death depends on nuclear localization and functional analysis of putative PvAvh74 *N-*glycosylation site mutants. **(A)** PvAvh74-GFP fusion protein transiently expressed in *N. benthamiana* protoplasts by PEG-mediated transformation. Confocal microscopy images show the subcellular localization of PvAvh74^NES^ and PvAvh74^nes^. A plasmid directing expression of the *Arabidopsis thaliana* nuclear protein derivative AtHY5-mCherry was co-transformed into the protoplasts. From left to right: GFP (488 nm), mCherry (587 nm), chloroplast autofluorescence, merged, bright-field (BF); bar = 10 μm. **(B)** GFP, PvAvh74^NES^, and PvAvh74^nes^ were transiently expressed in *N. benthamiana* leaves, and images were captured at 6 dai. **(C)** Ion leakage from cell death triggered by PvAvh74^NES^ and PvAvh74^nes^. Data are means ± SE based on three independent replicates (Student’s *t*-test, ^∗∗^*P* < 0.01). **(D)** Leaf of *N. benthamiana* showing typical response 6 dai with PvAvh74 (lower right region of leaf) or two putative *N*-glycosylation mutants, PvAvh74^N22A^, and PvAvh74^N465A^ (upper left and upper right, respectively). Infiltration with GFP (lower left) was carried out as a negative control. **(E)** Western blot analysis of extracts from *N. benthamiana* leaves transiently expressing PvAvh74-HA, GFP-HA, PvAvh74^N22A^-HA, or PvAvh74^N465A^-HA, using anti-HA monoclonal antibody. **(F)** Ion leakage (%) measurement of infiltration sites. Data are means ± SE based on three independent replicates (Student′s *t*-test, ^∗∗^*P* < 0.01).

PvAvh74 also contains a nuclear localization signal (NLS)-like sequence at its C terminus. To determine if this sequence is required for its cell death activity, we fused the GFP protein to the C terminus of PvAvh74 and expressed this in *N. benthamiana* protoplasts. As a positive control, the nuclear-localized protein AtHY5, fused with the mCherry marker, was co-expressed with the tagged PvAvh74 protein. After PEG-mediated transient transformation, the PvAvh74 signal (GFP) overlapped with the AtHY5 signal (mCherry), suggesting that PvAvh74 is localized to the nucleus (Figure 2A). To determine whether PvAvh74-dependent cell death requires its nuclear localization, we fused PvAvh74 with an artificial *NES* or a dysfunctional derivative (*nes*) at its carboxyl terminus. As anticipated, PvAvh74^NES^ was exported from the nucleus and showed fluorescence in the cytoplasm, whereas PvAvh74^nes^ was confined to the nucleus (Figure 2A). Then, we transiently expressed PvAvh74, PvAvh74^NES^, and PvAvh74^nes^ in *N. benthamiana* leaves. We found that expression of PvAvh74 or PvAvh74^nes^ induced cell death, whereas expression of PvAvh74^NES^ did not (Figure 2B). Ion leakage at infiltrated sites (Figure 2B) was measured at 6 dai (Figure 2C) to quantify cell death. These results indicate that PvAvh74-induced cell death depends on its nuclear localization.

### Functional Analysis of Two Putative *N*-Glycosylation Mutants in PvAvh74

Effector proteins and other secreted proteins can be glycosylated to facilitate their trafficking in the apoplast or within host cells. Indeed, an *N-*glycosylated effector, ALG3, was reported to be necessary for *Magnaporthe oryzae* to evade host innate immunity (Chen et al., 2014). PvAvh74 contains two predicted *N-*glycosylation sites, Asn-22 (NQTE) and Asn-465 (NLSK). In order to determine whether these sites were necessary for PvAvh74-induced necrosis, we created Asn-to-Ala mutations at both sites, and the mutants, PvAvh74^N22A^ and PvAvh74^N465A^, were expressed in *N. benthamiana* leaves. PvAvh74, PvAvh74^N22A^, and PvAvh74^N465A^ were all stably expressed in *N. benthamiana* leaves, with approximately the same molecular mass (53 kDa), as measured by SDS-PAGE (Figure 2E). Quantification of cell death was measured by electrolyte leakage (Figure 2F). Interestingly, the two mutants did not cause cell death at the infiltration sites (Figure 2D). Additionally, the location of PvAvh74, PvAvh74^N22A^, and PvAvh74^N465A^ were checked by confocal microscopy. We found that PvAvh74^N22A^ did not exclusively localize to the nucleus, while PvAvh74^N465A^ still localized to the nucleus (Supplementary Figure S5). These results suggested that both of these putative *N*-glycosylation sites are crucial for PvAvh74 to induce necrosis in *N. benthamiana*. The location of PvAvh74^N465A^ suggested that this putative *N*-glycosylation site is required to induce cell death but not essential for localization.

**FIGURE 3:**
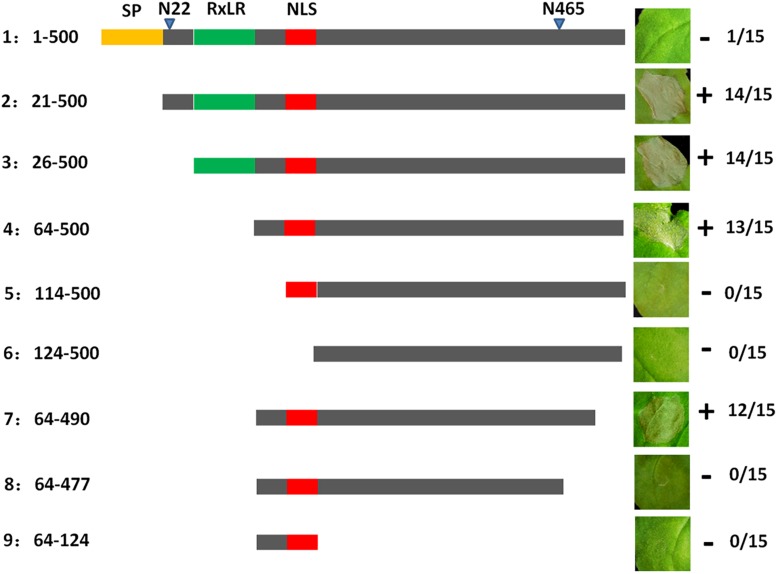
Deletion analysis of PvAvh74. Plasmids encoding deletion mutants of PvAvh74 were infiltrated into *N. benthamiana* leaves to determine which regions of PvAvh74 were required for necrosis. A depiction of the PvAvh74 protein and derivatives is shown as a horizontal gray bar, with the signal peptide (SP) in yellow, RxLR motif in green, and nuclear localization sequence (NLS) in red. The inset photographs show typical response to infiltration with each construction on leaves of *N. benthamiana*, 6 dai. Plus (+) indicates that cell death resulted, while minus (-) indicates absence of cell death. The fraction numbers at far right indicate the number of infiltrated sites where cell death was observed/total number of infiltrated sites. Assays were repeated three times with similar results.

To further analyze the function of PvAvh74, we engineered nine mutants deleting various regions of the PvAvh74 protein, and evaluated their ability to induce cell death. The full-length (500 amino acids) PvAvh74 contained an intact signal peptide, RxLR motif, and NLS did not trigger cell death. In contrast, deletion mutants that removed the amino terminus, including the signal peptide and RxLR motif, as well as 10 amino acids at the C terminus, were able to induce cell death (Figure 3).

### PvAvh74 Transient Expression Induces H_2_O_2_ Accumulation and Enhances *N. benthamiana* Resistance to *P. capsici*

**FIGURE 4:**
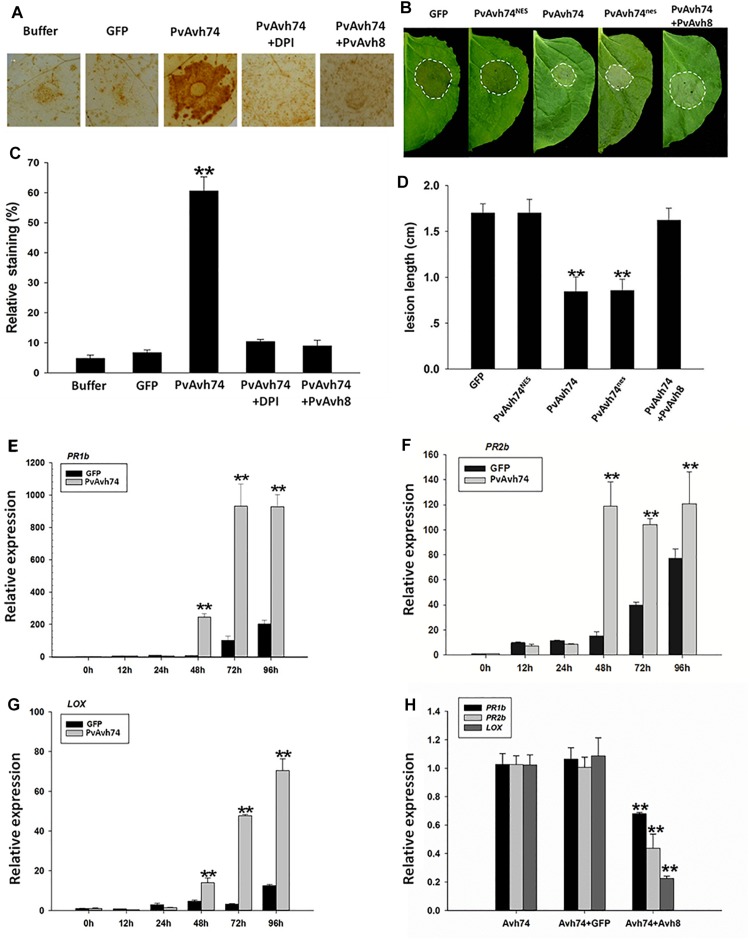
Transient expression of PvAvh74 induces H_2_O_2_ accumulation and enhances *N. benthamiana* resistance to *Phytophthora capsici*. **(A)** DAB staining of *N. benthamiana* leaves expressed with GFP, PvAvh74, PvAvh74+DPI, or PvAvh74+PvAvh8. **(B)** Lesions on *N. benthamiana* leaves expressing GFP, PvAvh74^NES^, PvAvh74^nes^, or PvAvh74+PvAvh8 inoculated with *P. capsici* at 36 hpi. **(C)** Quantification of staining shown in **(A)**. **(D)** Lesion diameter on *N. benthamiana* leaves measured 36 hpi. Data are means ± SE based on three independent replicates (Student’s *t*-test, ^∗∗^*P* < 0.01). Expression levels of *PR1b* **(E)**, *PR2b* **(F)**, and *LOX* **(G)** genes in *N. benthamiana* induced by PvAvh74 at different time points. **(H)** Relative expression levels of *PR1b*, *PR2b*, and *LOX* genes induced by expressing PvAvh74, PvAvh74+GFP, and PvAvh74+PvAvh8. The relative expression levels of defense-related genes were detected at 3 dai. Data are means ± SE based on three independent replicates (Student’s *t*-test: ^∗∗^*P* < 0.01).

To identify whether PvAvh74 plays an important role in ROS accumulation, we evaluated H_2_O_2_, levels by DAB staining. DPI, a NADPH oxidase inhibitor, was used to test whether PvAvh74-induced cell death requires an oxidative burst. When agroinfiltrated with PvAvh8, another RxLR effector from the same *P. viticola* isolate, PvAvh74 did not cause cell death (Supplementary Figures S6A,B). In addition, *PvAvh74* and *PvAvh8* were highly expressed at 48 hpi during *P. viticola* infection (Supplementary Figures S6C,D). DAB staining of PvAvh74-expressing leaves revealed reddish-brown foci, but these were not observed when DPI was used or when PvAvh8 was co-expressed (Figure 4A). In addition, the relative level of DAB staining of PvAvh74-expressing leaves was much higher than that for GFP, PvAvh74+DPI, or PvAvh74+PvAvh8 (Figure 4C). These results suggested that PvAvh74 could induce H_2_O_2_ accumulation.

To determine whether PvAvh74 plays a role in plant immunity, low concentrations of *Agrobacterium* suspensions (OD_600_ = 0.1) were used to avoid rapid cell death. Two days after infiltration, leaves treated with PvAvh74, GFP, PvAvh74^NES^, and PvAvh74^nes^ were inoculated with ∼10 μl of *P. capsici* zoospores suspension, corresponding to ∼100 zoospores. Then, lesion diameters were measured at 36 hpi. Lesion diameters on *N. benthamiana* leaves infiltrated with PvAvh74^NES^ were similar to those from GFP and PvAvh74+PvAvh8 (Figure 4D), whereas lesion diameters of PvAvh74^nes^ and PvAvh74 were significantly smaller (Figure 4B). These results indicated that nuclear localization is required for PvAvh74 recognition, which induces plant defenses.

### PvAvh74 Expression Enhances Defense- Associated Genes in *N. benthamiana*

To confirm that PvAvh74 is involved in plant defense responses, the expression of three defense-associated genes, *PATHOGENESIS-RELATED PROTEIN PR1b*, *PR2b*, and *LIPOXYGENASE* (*LOX*), was monitored in *N. benthamiana* leaves transiently expressing PvAvh74. *PR1b/2b* participate in salicylate-mediated signaling (Lee et al., 2013), while *LOX* is correlated to jasmonate-mediated signaling (Wang et al., 2000). All these genes play vital roles in downstream disease defense. The results show that after transient expression of PvAvh74, *PR1b/2b*, and *LOX* were upregulated (Figures 4E–G). In addition, PvAvh74 recognition-promoted expression of defense genes can be suppressed by PvAvh8 (Figure 4H). These results revealed that PvAvh74 recognition promotes expression of defense-associated genes in *N. benthamiana*.

### Cell Death Induced by PvAvh74 Requires SGT1, Hsp90, RAR1, NDR1, and EDS1, but Not BAK1

**FIGURE 5:**
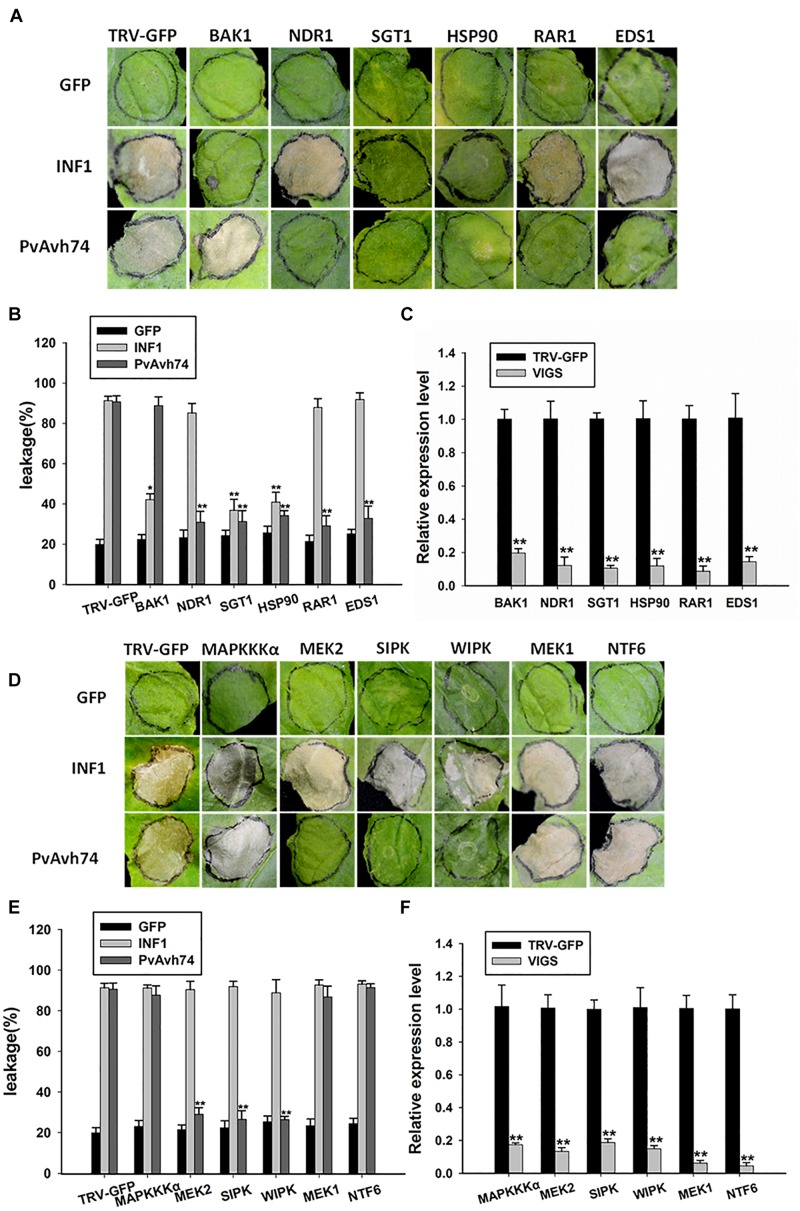
Transient expression of PvAvh74 in TRV-VIGS-silenced *N. benthamiana* leaves. **(A,D)** Transient expression of PvAvh74 in *N. benthamiana* leaves in which SGT1, Hsp90, RAR1, EDS1, NDR1, MEK1, MEK2, MAPKKKα, NTF6, WIPK, SIPK, or BAK1 had been silenced. GFP and INF1 were used as controls. Leaves showing typical effects were photographed 6 days later. The VIGS assay was repeated three times using five plants for each silenced gene. **(B,E)** Ion leakage (%) measurement at infiltration sites. **(C,F)** Quantitative RT-PCR analysis of expression levels of multiple genes in *N. benthamiana* relative to control plants. Data are means ± SE based on three independent replicates (Student’s *t*-test: ^*^P < 0.05 and ^∗∗^*P* < 0.01).

Plant intracellular immune receptors are generally characterized as nucleotide-binding and leucine-rich repeat receptors (NLRs), which are further classified according to the N-terminal domain as CC (coiled-coil) or TIR (Toll/Interleukin-1 receptor). Generally, the EDS1 (Enhanced disease susceptibility 1) and NDR1 (Non-race-specific disease resistance 1) proteins are required for the function of TIR-NLR and CC-NLR R proteins, respectively (Aarts et al., 1998). Moreover, Suppressor of G-two allele of Skp1 (SGT1), Mla12 resistance (RAR1), and heat shock protein 90 (HSP90) function as an R protein complex and are reported to facilitate the HR mediated by many NLRs (Kadota et al., 2010). In addition, the receptor-like kinase BAK1/SERK3 is known as a major modulator of PTI, as silencing of BAK1/SERK3 could reduce the cell death induced by INF1 (Chaparrogarcia et al., 2011). To test whether SGT1, Hsp90, RAR1, EDS1, or NDR1 is involved in PvAvh74-induced cell death, we carried out TRV-VIGS assays to silence SGT1, Hsp90, RAR1, EDS1, and NDR1 in *N. benthamiana*. qRT-PCR results showed that expression of the respective target genes was significantly reduced (Figure 5C). Subsequently, GFP, PvAvh74, and INF1 were expressed in those silenced plants. We found that PvAvh74 could not induce cell death in SGT1, Hsp90, RAR1, EDS1, and NDR1-silenced plants though PvAvh74 was expressed in those silenced plants (Figures 5A,B and Supplementary Figure S7). However, PvAvh74-induced cell death was observed in BAK1/SERK3 silenced plants (Figure 5A). These results indicated that cell death triggered by PvAvh74 in *N. benthamiana* leaves depends on SGT1, Hsp90, RAR1, EDS1, and NDR1, but not BAK1.

### Cell Death Induced by PvAvh74 Is Associated With Mitogen-Activated Protein Kinase Cascades

Mitogen-activated protein kinase cascade pathways play critical roles in plant immunity signaling during PTI and ETI (Pitzschke et al., 2009). To determine whether MAPK kinases are involved in PvAvh74-induced cell death, the MAP kinases MAPKKKα, MEK1, MEK2, and NTF6, as well as the other two important MAPKs, wound-induced protein kinase (WIPK) and salicylic acid-induced protein kinase (SIPK) (Asai et al., 2008), were silenced in *N. benthamiana* using TRV-VIGS. qRT-PCR analysis showed that expression of these genes was reduced significantly compared with that of TRV-GFP (Figure 5F). Subsequently, we expressed GFP, PvAvh74, and INF1 in the silenced plants. The results showed that PvAvh74-induced cell death in *N. benthamiana* requires MEK2, SIPK, and WIPK but not MAPKKKα, MEK1, and NTF6 (Figures 5D,E).

## Discussion

Effectors are essential for pathogens to modulate host immunity. Some of these effectors are reported to elicit cell death, which is a typical avirulence determinant. For example, the late blight *R* gene of *Solanum demissum*, R1, specifically recognizes the avirulence (*Avr*) gene AVR1 from a *P. infestans* isolate (van der Lee et al., 2004; Ballvora et al., 2010). These avirulence genes recognized by specific *R* genes causing cell death is in accord with the “gene-for-gene” model. However, some avirulence homolog (*Avh*) genes cause cell death where the R gene remains unidentified, such as Avh241 (Yu et al., 2012) and Avh238 (Yang et al., 2017) in *P. sojae*, or PITG_22798 (Wang et al., 2017) in *P. infestans*.

In the present study, a candidate RxLR effector from *P. viticola*, PvAvh74, was also characterized as an elicitor to cause necrosis in *N. benthamiana*. However, PvAvh74 cannot induce cell death in grapevine (*V. vinifera*). This evidence is consistent with three *P. viticola* RxLR effectors, PVITv1021061, PVITv1008294, and PVITv1008311 (Brilli et al., 2018). The possible reason for this phenomenon may be that grapevine (*V. vinifera*) cannot recognize *P. viticola* effectors to initiate a immune response like cell death. In addition, a similar result was reported by Wang et al. (2017) that an RxLR effector PITG_22798 from *P. infestans* triggers cell death in *N. benthamiana* but not in potato species. The cell death triggered by PvAvh74 may depend directly or indirectly on unknown R proteins in *N. benthamiana*. Using TRV-VIGS, 12 genes associated with plant defense or MAPK cascades were silenced in *N. benthamiana*. The results show that PvAvh74-triggered cell death requires SGT1, Hsp90, RAR1, EDS1, and NDR1 but not BAK1. SGT1, Hsp90, and RAR1 are reported to function as an R protein complex that plays a role in stabilizing the NLRs (Kadota et al., 2010). EDS1 and NDR1, two important resistance factors, are generally independently required for R gene-mediated signaling pathways (Aarts et al., 1998). However, in this study, both EDS1 and NDR1 were found to be involved in PvAvh74-triggered cell death. This result is in agreement with a study by Peng et al. (2003) showing that both EDS1 and NDR1 are required for the development of resistance in *Arabidopsis* treated with harpin.

Furthermore, our findings link PvAvh74 with MAPK cascades. MEK2, SIPK, and WIPK are involved in PvAvh74-triggered cell death. MEK2 plays a vital role in plant immunity defense and is known to act upstream of salicylic acid to induce expression of SIPK and WIPK (Takahashi et al., 2007; Asai et al., 2008). MAPK cascades play a vital role in plant defense and participate in ROS accumulation (Asai et al., 2008; Adachi et al., 2015). Interestingly, the results of DAB staining showed that H_2_O_2_ accumulated in *N. benthamiana* leaves expressing PvAvh74. Taken together, these results suggest that PvAvh74-triggered cell death may depend on ETI, perhaps by recognizing R proteins in tobacco directly or indirectly, for instance, SGT1, Hsp90, RAR1, EDS1, and NDR1 in *N. benthamiana*. Additionally, PvAvh74-triggered cell death requires MEK2. As downstream effectors of MEK2, WIPK, and SIPK participate in PvAvh74-triggered cell death. We hypothesize that as a result of MAPK cascades, H_2_O_2_ ultimately accumulated in PvAvh74-expressing leaves. Although effectors are generally thought to suppress plant immunity or are recognized as avirulence genes resulting in ETI, some studies suggest that effectors have varied functions. For example, PvRxLR16 (Xiang et al., 2017) and CRN161 (Rajput et al., 2015) have been characterized as inducers of plant immunity. In this study, recognition of PvAvh74 induced defense responses to *P. capsici* in *N. benthamiana*, and several marker genes correlated to JA and SA were upregulated. Therefore, PvRxLR74-induced plant defense to *P. capsici* may correlate with ROS accumulation.

Many reports have shown that the nucleus plays an important role during the interaction between effectors and their hosts. In *H. arabidopsidis*, 66% of encoded HaRxLRs were found to be targeted to the nucleus and cytoplasm, or the nucleus specifically (Caillaud et al., 2012). Recently, a live-cell imaging study of 76 RxLR effectors from *P. viticola* showed that, in tobacco plants, 63 effectors were trafficked to either the nucleus and cytoplasm, or the nucleus specifically (Liu et al., 2018). In this study, by fusing PvAvh74 with GFP, we showed that PvAvh74 was a nuclear-localized protein. By using a NES sequence, PvAvh74^NES^ could be relocated to the cytoplasm. In addition, the functions of PvAvh74 were modified, as the lesions in PvAvh74^NES^ were larger than in PvAvh74, and PvAvh74^NES^ did not induce cell death at infiltrated sites. Similar observations revealed that nuclear localization is essential for Avh238 and PITG_22798 to trigger cell death in *N. benthamiana*.

The signal peptide is an important feature of effector proteins, and can be used to identify potential effectors in proteome data. In general, there are two distinct domains in RxLR effectors, N-terminal and C-terminal. The signal peptide and RxLR motif at the N terminus was reported to target the mature protein in the host cells. The effector activity is localized at the C-terminal region, and this region encodes the effector domain (Kamoun, 2006).

In a recent study, 49 (excluding PvAvh74) candidate RxLR effector signal peptides in *P. viticola* were found to be functional using the yeast signal sequence trap assay (Liu et al., 2018). In this study, the predicted signal peptide of PvAvh74 was also found to be functional. In addition, the full-length PvAvh74 protein, containing the signal peptide, could not cause necrosis in *N. benthamiana.* This result was similar with results from a study by Bos et al. (2006) showing that a full-length RxLR effector, AVR3a^KI^, containing a signal peptide cannot suppress cell death induced by INF1. Deletion analysis indicated that the C-terminal 427 amino acids of PvAvh74, without the RxLR motif, are sufficient to cause necrosis. This result is consistent with findings of Bos et al. (2006) that the C terminus of AVR3a^KI^ without the RxLR motif is sufficient for avirulence and suppression functions.

Recently, 10 out of 83 predicted PvRxLR effectors from a Chinese *P. viticola* isolate genome were found to cause necrosis in *N. benthamiana* leaves (Liu et al., 2018). In another study, PvRxLR16 was characterized as an elicitor to cause necrosis in *N. benthamiana* leaves (Xiang et al., 2017). Notably, 17 RxLR effectors from the same *P. viticola* isolate could suppress PvRxLR16-induced cell death in *N. benthamiana* (Xiang et al., 2016). In this study, PvAvh74-triggered cell death could be suppressed by another effector, PvAvh8, from the same isolate. This result is consistent with the observation that PvRxLR16, when co-expressed with PvRxLR1, did not trigger cell death or immune responses in tobacco plants. Actually, effector-induced cell death can be suppressed even by variants of the effector itself; for example, four “no cell death” variants of PcCRN83_152 could suppress PcCRN83_152-mediated cell death (Amaro et al., 2018). Therefore, we speculate that there are interactions among effectors in the same pathogen isolate, suggesting that an isolate of *P. viticola* may not induce cell death or immune responses in grapevine if PvAvh74 and PvAvh8 coexist. To our knowledge, *P. viticola* still cannot be artificially cultured. Therefore, it is difficult to over-express or silence PvAvh74 in *P. viticola* during infection. Despite this, PvAvh74 may be used to accelerate the identification of defense genes participating in resistance to *P. viticola*. Defense induction occurs because the non-host plant *N. benthamiana* likely contains an R gene that is able to recognize PvAvh74. The next step will be to screen the targets of PvAvh74 in grapevine, and this study provides a clue to further investigate the functions of PvAvh74 associated with host immunity.

## Data Availability

The raw data supporting the conclusions of this manuscript will be made available by the authors, without undue reservation, to any qualified researcher.

## Author Contributions

YX conceived the study. XY and BS conducted the experiments. MD, TC, GX, and YL contributed to tobacco plant cultivation and confocal microscopy. XY wrote the manuscript. RL, GL, and YX revised the manuscript.

## Conflict of Interest Statement

The authors declare that the research was conducted in the absence of any commercial or financial relationships that could be construed as a potential conflict of interest.
