# Supplementary material for: The Nuclear-Localized RxLR Effector PvAvh74 From Plasmopara viticola Induces Cell Death and Immunity Responses in Nicotiana benthamiana
Source: Front Microbiol. 2019 Jul 10;10:1531. doi: 10.3389/fmicb.2019.01531 (PMC6636413; doi:10.3389/fmicb.2019.01531)
Supplement: Supplementary file 1 [file Data_Sheet_1.docx]

Supplementary Material


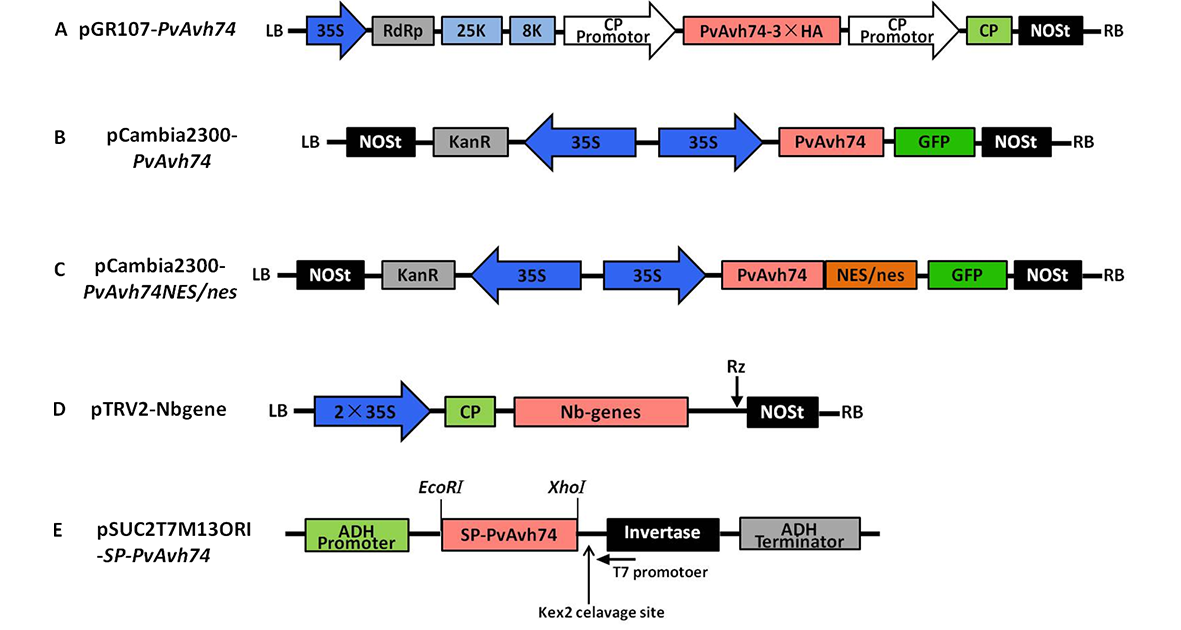


**Fig S1. Details of vector constructions used in this study.**

**(A)** For the PVX assay, PvAvh74 without the predicted signal peptide and PvAvh74 deletion mutant were ligated into pGR107 vector with *Sma* I and *Not* I restriction enzymes sites. **(B)** and **(C)** For subcellular location, PvAvh74, PvAvh74^NES^ and PvAvh74^nes^ were ampliﬁed using appropriate restriction enzymes and ligated into pCAMBIA2300-GFP vector. **(D)** For Virus-Induced Gene-Silencing (VIGS) assay, fragments of the target genes were ampliﬁed then ligated into pTRV2 vector with *EcoR* I and *BamH* I restriction enzymes sites. **(E)** To confirm the secretion function of PvAvh74 signal peptide, the predicted signal peptide sequence of PvAvh74 was cloned containing *EcoRI* and *XhoI* restriction sites and then introduced into pSUC2T7M13ORI (pSUC2). **CP:** coat protein, **NOSt:** nopaline synthase terminator, **RdRp:** RNA dependent RNA polymerase, **35S:** Cauliflower mosaic virus 35S promoter, **SP:** signal peptide, **LB:** left T-DNA border, **RB:** right T-DNA border, **Rz:** self-cleaving ribozyme, **KanR:** neomycin phosphotransferase gene (NPT II), **GFP:**  green fluorescent protein.


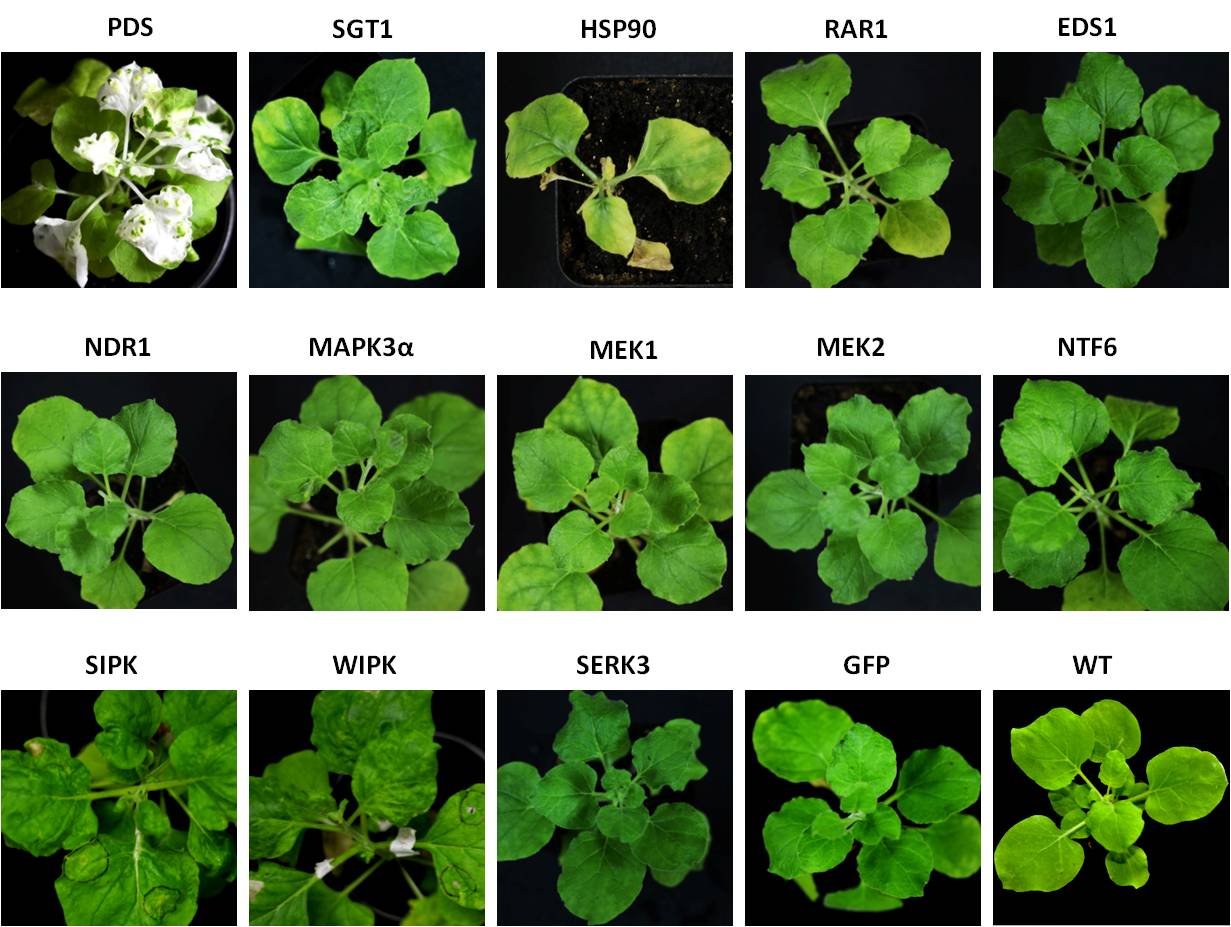


**Fig S2. Morphology of *N. benthamiana* plants with silenced genes by TRV-VIGS.** *Agrobacterium* carrying TRV1 and TRV2 were co-inoculated by using a needleless syringe onto the abaxial side of leaves of 3-week-old plants. The plants were photographed at 3 weeks after inoculation. Technique control of VIGS assay was carried out by silence the *NbPDS*. TRV2: *GFP* was used as a vector control.

**
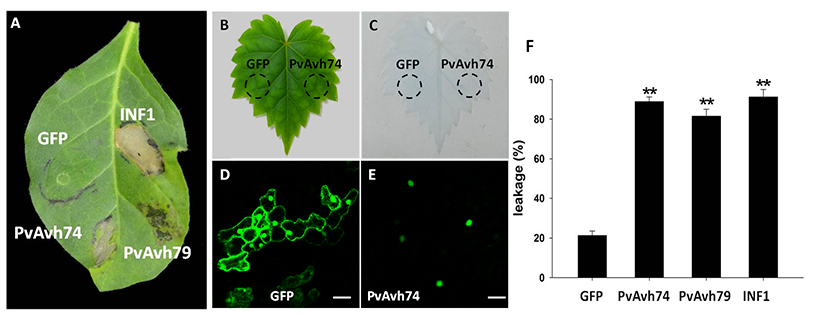
**

**Fig S3. PvAvh74 can induced cell death in *N. tabacum* but not trigger cell death in *Vitis vinifera* leaves.**

Leaves of *N. tabacum* and grape *in vitro* (*V*. *vinifera* susceptible cultivar Thomson seedless) were infiltrated with *Agrobacterium tumefaciens* carrying PvAvh74. **(A)** PvAvh74 induced cell death in *N. tabacum* leaves, INF1 and PvAvh79 (An unpublished RxLR effectors ) were used as positive control. **(B)** and **(C)** PvAvh74 can not trigger cell death in leaves of *V*. *vinifera* susceptible cultivar Thomson seedless. Expression of GFP **(D)** and PvAvh74-GFP **(E)** were examined by confocal microscopy in grape leaves. **(F)** Relative leakage from the infiltrated *N. tabacum* leaf discs was measured as a percentage of leakage from boiled discs. Photographs were taken at 7 dai for *N. tabacum* and grape leaves. The experiment was repeated three times with similar results. Data are the means of three independent experiments and the error bars represent ± SE. Asterisks indicate significant differences from the control. (Student’s t test; ** P<0.01). Bar, 25μm.

**
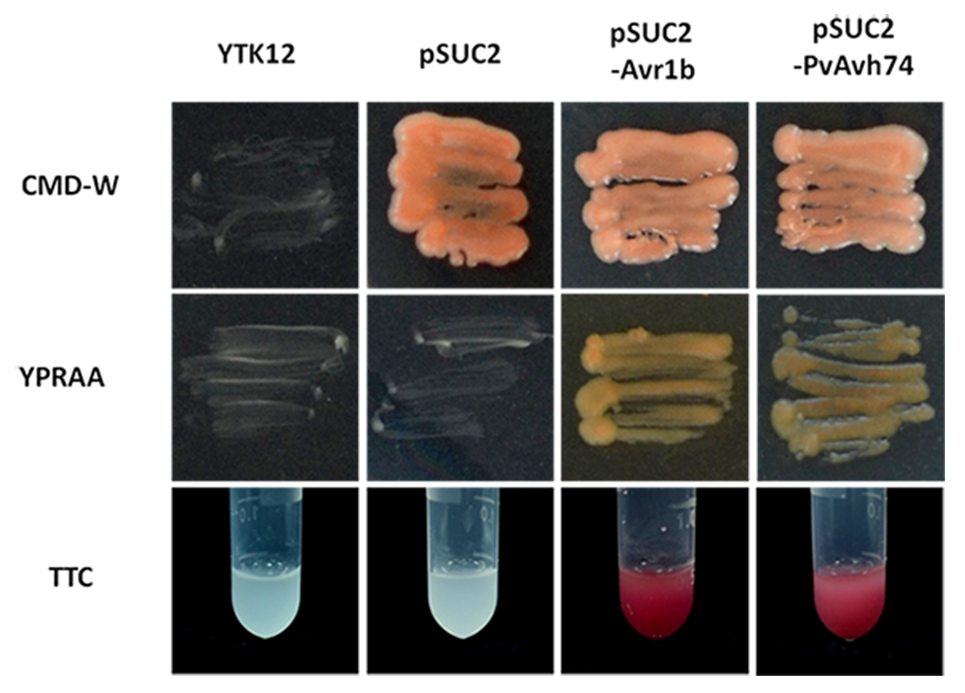
**

**Fig S4. Functional validation of the signal peptide of PvAvh74 using the yeast invertase secretion assay.**

Yeast strain YTK12 carrying the pSUC2 vector is able to grow on CMD-W medium (lacking Trp), but not on YPRAA medium. YTK12 carrying pSUC2-Avr1b or pSUC2-PvAvh74 is able to grow on YPRAA medium. The invertase secretion test is based on the conversion of TTC into red-colored triphenylformazan. YTK12 and YTK12 carrying the empty pSUC2 vector were the negative controls, while YTK12 carrying the pSUC2-Avr1b was a positive control.


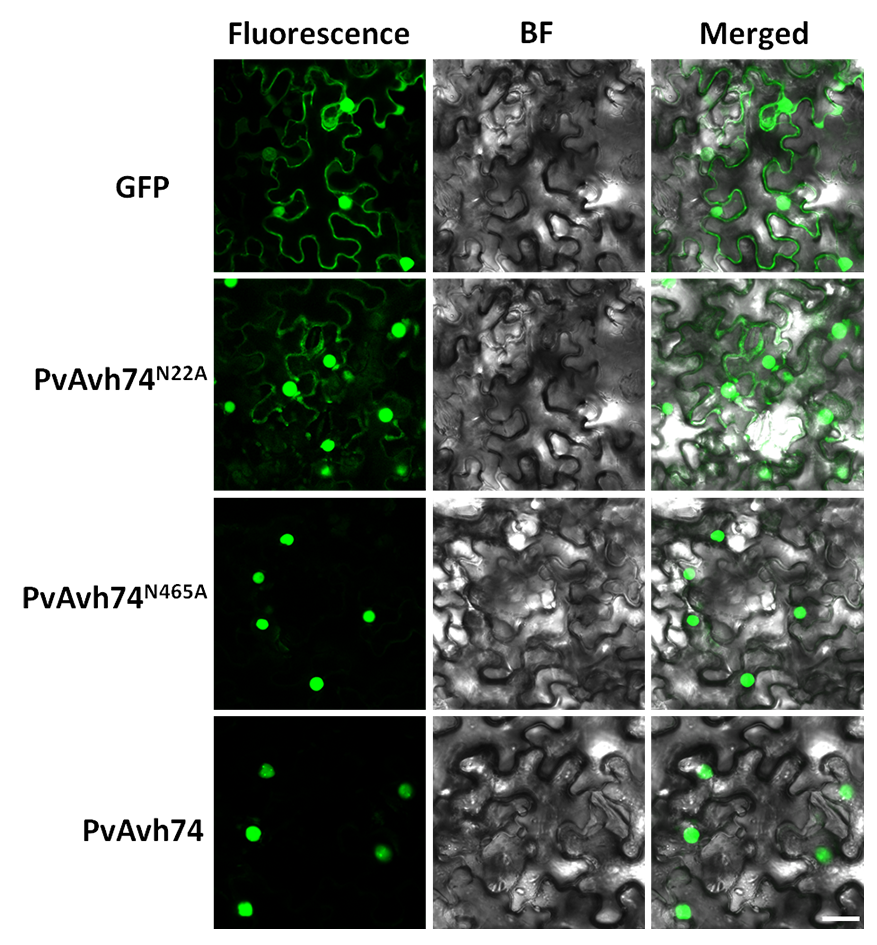


**Fig S5. Localization of PvAvh74, PvAvh74^N22A^ and PvAvh74^N465A^.** The mutants PvAvh74^N22A^ , PvAvh74^N465A^, and PvAvh74, were constructed into pCAMBIA2300-GFP vector. PvAvh74, PvAvh74^N22A^, and PvAvh74^N465A^ were transiently expressed in *N. benthamiana* leaves using agroinfiltration. Two days later, fluorescence were examined using confocal microscopy. Mutant PvAvh74^N22A^ did not exclusively localize to the nucleus. Bar, 25 µm.


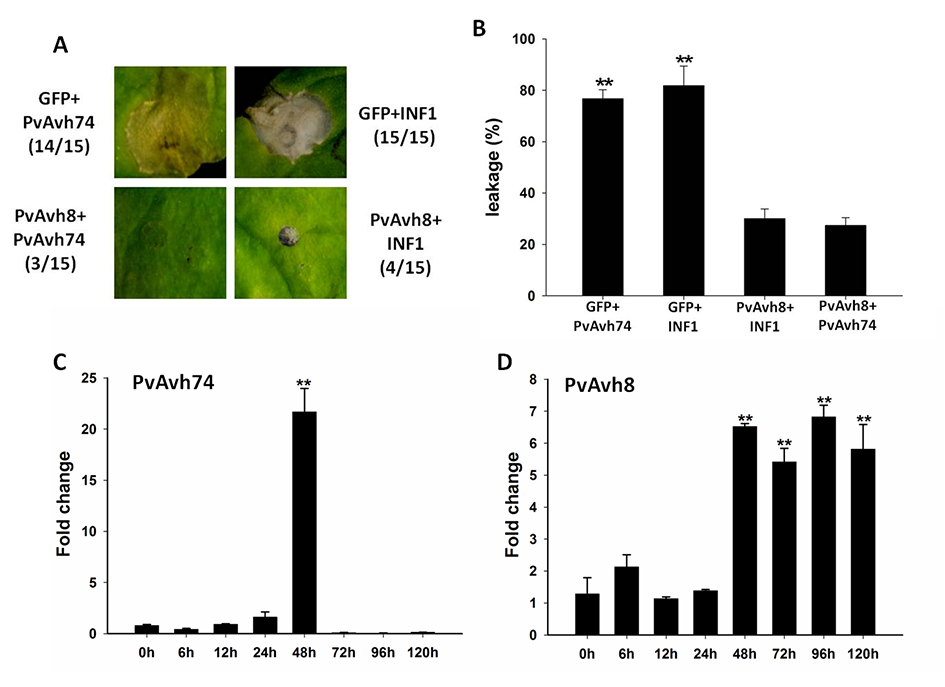


**Fig S6.  *PvAvh74* and *PvAvh8* are induced during *P. viticola* infection and PvAvh74**-**induced cell death can be suppressed by PvAvh8 in *N. benthamiana.***

**(A)** PvAvh74 and INF1 induced cell death can be suppressed by PvAvh8 in *N. benthamiana*. Bracket: (Number of cell death / Number of all the infiltrated sites). **(B)** Relative leakage from the infiltrated leaf discs was measured. The leaf discs of *V. vinifera* susceptible cultivar Pinot Noir were drop-inoculated with the sporangia suspension of *P. viticola* and harvested at indicated time-points post inoculation. The *PvActin* gene was used as an internal control. **(C)** *PvAvh74* was highly expressed at 48 hpi then declined at the other infection stages. **(D)** *PvAvh8* was highly expressed at 48 hpi and the expression persisted during the subsequent infection stages. Data are the means of three independent experiments and the error bars represent ± SE. Asterisks indicate significant differences from the control (Student’s t test, ** P<0.01).


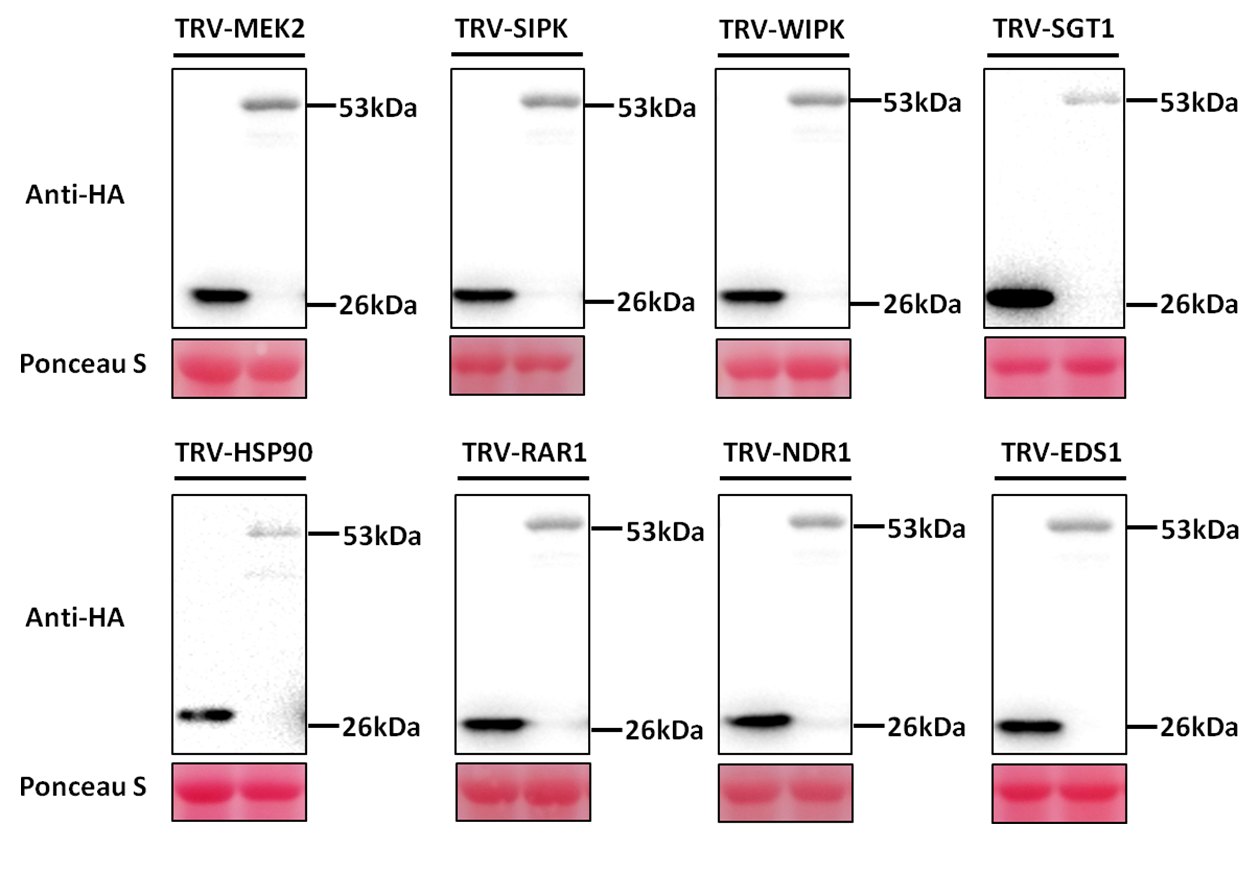


**Fig S7. Western blotting analysis of PvAvh74 from gene-silenced *N. benthamiana* leaves.**

PvAvh74-HA was transiently expressing in *MEK2*, *SIPK*, *WIPK*, *SGT*, *Hsp90*, *RAR1*, *EDS1*, and *NDR1* silenced *N. benthamiana* leaves. Western blotting analysis of PvAvh74 were carried out from gene-silenced *N. benthamiana* leaves using anti-HA monoclonal antibody.
